# Feasibility and efficacy of implementing group visits for women’s health conditions: a systematic review

**DOI:** 10.1186/s12913-023-09582-6

**Published:** 2023-05-26

**Authors:** Sophia Gerontakos, Matthew Leach, Amie Steel, Jon Wardle

**Affiliations:** 1 National Centre for Naturopathic Medicine, Southern Cross University, A Block, Military Road, Lismore, NSW 2480 Australia; 2 Faculty of Health, University of Technology Sydney, Australian Research Centre in Complementary and Integrative Medicine, Broadway, NSW Australia

**Keywords:** Shared medical appointments, Group visits, Group medical visits, Women’s health, Female reproductive health, Systematic review, Chronic pelvic pain, Polycystic ovary syndrome, Gynaecological cancer, Breast cancer

## Abstract

**Background:**

Shared medical appointments, also known as group visits, are a feasible and well-accepted approach for women receiving antenatal care, yet the feasibility and efficacy of this approach for female-specific reproductive conditions is uncertain.

**Objective:**

The aim of this systematic review was to (a) determine the feasibility of group visits in adults with any female-specific reproductive condition, and (b) identify whether delivering group care for these conditions impacts clinical outcomes.

**Method:**

Six databases and two clinical trials registries were searched from inception through to 26 January 2022 for original research examining group medical visits or group consultation interventions for adults with female reproductive conditions or pathologic conditions specific to the female reproductive system.

**Results:**

The search yielded 2584 studies, of which four met the inclusion criteria. Included studies sampled women with breast cancer, chronic pelvic pain, polycystic ovary syndrome and gynaecological cancers. Studies reported high levels of patient satisfaction, with participants indicating their expectations had been met or exceeded. The impact of group visits on clinical outcomes was inconclusive however.

**Discussion/conclusions:**

The studies in this review indicate delivery of female-specific healthcare via a group model maybe feasible and well-accepted. The review provides a solid basis for proposing larger and longer studies on group visits for female reproductive conditions.

**Trial registration:**

The review protocol was registered with PROSPERO (CRD42020196995).

**Supplementary Information:**

The online version contains supplementary material available at 10.1186/s12913-023-09582-6.

## Background

Since the emergence of group visits in the United States in the early 1990’s [1], the innovative medical delivery model has spread throughout the world. Group visits (also known as shared medical appointments [SMAs], group medical visits [GMVs] or integrative medicine group visits [IMGVs] where complementary therapies are included in the consultations [2]), are patient-centred models of care whereby a group of patients consult with one or more healthcare providers in a concurrent session [3]. Generally, group visits include a consultation with a medical doctor (within the group or privately), measurement of vital signs, medication review, patient education and in some cases, involvement of other allied health professions specific to the needs of the group [4]. Therefore, group visits include all the components of a one-on-one medical appointment along with patient education and peer support. For the purpose of this review, the term ‘group visit’ has been employed to describe any health-care intervention delivered in a group (that would typically be delivered via a one-on-one consultation), including SMAs, IMGVs and GMVs.

Current evidence indicates group visits may have a positive impact on health service delivery. In studies to date, group visits have been shown to (a) be more cost-effective than one-on-one consultations [5, 6], (b) increase accessibility to healthcare in diverse and/or underserved populations [7], (c) be well-received by patients and providers [5, 8], and (d) be effective in managing conditions such as diabetes [9, 10] and chronic pain [11, 12]. The group dynamic can provide additional benefits to patients where a sense of human connection and empowerment may be fostered alongside increased capacity for patient education [3, 13]. To date, no adverse effects have been associated with group-delivered health care [6]. Although several studies suggest patients attending group visits may receive more comprehensive care (than those attending one-on-one care), there may be disadvantages to the model. For example, some studies have favoured one-on-one care for the amount and quality of time spent with the practitioner (audiologist) [6]. Disadvantages reported by Australian providers have mainly been attributed to initial administrative issues which have been overcome at subsequent group visits [8].

In the area of women’s health, group-based antenatal care has been associated with improvements in the disclosure of information (i.e. speaking about issues that women typically would not have spoken about to a provider during one-on-one care) [14]. This disclosure may be facilitated by being in a safe space with other women with similar concerns, and feeling more empowered and self-confident [14]. Group-based antenatal care also has been found to be feasible, more effective and affordable than individual care in a variety of settings and populations [15–17]; and is associated with a reduced risk of premature birth, lower incidence of low birth weight infants, and reduced health care expenditure [18]. In addition, a high level of patient satisfaction has been reported among women receiving group-based antenatal/prenatal care [19, 20]. Similarly, in women with gestational diabetes mellitus (GDM) or pre-GDM, group-based prenatal care has been associated with significantly lower rates of progression from pre-GDM to GDM, lower rates of insulin prescription [21]; and found to be more cost-effective than individual prenatal care [22].

Evidence supporting the effectiveness and feasibility of group visits in the area of maternal health suggests a need and desire for women to participate in group-delivered healthcare. Female reproductive conditions such as endometriosis, polycystic ovary syndrome (PCOS) and chronic pelvic pain are increasingly common in women of reproductive years. Endometriosis for example, has a similar prevalence as diabetes worldwide [23], and yet evidence indicates mainstream medicine is not meeting the healthcare needs of those living with the condition [24]. Given the chronic nature of many female-specific conditions (for example endometriosis [25] and PCOS [26]), and the evidence in support of group-based maternal care and SMAs for chronic disease management, there is a sound rationale for investigating the effectiveness of SMAs for female-specific conditions.

Although SMAs are an effective model for delivering healthcare to patients with chronic pain and diabetes, as well as expectant mothers, the impact of the model on women and gender-diverse people experiencing female-specific reproductive conditions is currently uncertain. The objective of this systematic review was to determine the feasibility, acceptability and clinical effectiveness of group visits in adults with any female-specific reproductive condition. In doing so, the review investigates the evidence-base for a potentially untapped model of care that may help improve health outcomes for people living with female reproductive conditions. As well as informing clinical practice, the findings from this review help identify important directions for future research examining the use of group visit models of care for the management of female reproductive conditions.

## Methods

This systematic review was conducted in accordance with the Preferred Reporting Items for Systematic Reviews and Meta-Analyses (PRISMA) guidelines [27]. The PRISMA checklist can be viewed in Supplementary file 1. A database search was conducted to identify peer-reviewed original research that fulfil the following inclusion criteria: examining SMAs, or group-based medical interventions (i.e. adaptations of the SMA model) for adult patients with female reproductive conditions or pathologic conditions specific to the female reproductive system. Although not a target population of this review, people identifying as gender diverse met the inclusion criteria. For the purposes of this review, SMAs were defined as any health-care intervention delivered in a group (that would typically be delivered via a one-on-one consultation) – this included SMAs, IMGVs and GMVs. Studies examining interventions that are routinely delivered in groups (for example yoga or group therapy) were excluded. The review examined two main outcomes: 1) efficacy (defined as any clinical outcome including health-related quality of life, change in general health status, self-efficacy, emotional health and attitudes, change in disease severity), and 2) feasibility (measured by cost-effectiveness, demand, patient satisfaction, provider satisfaction, implementation) of SMAs for adults with any female-specific reproductive condition.

The search strategy employed keywords and MeSH terms adapted from common terminologies used in existing literature, the OVID resource centre verified filter for women’s health [28] and previously used literature searches [29, 30]. The search included electronic databases (Scopus [Elsevier], MEDLINE [Ovid], EMBASE [Ovid], CINAHL [Ovid], AMED [Ovid] and Google Scholar as a supplementary search limited to first 10 pages) and two clinical trials registries (Australian New Zealand Clinical Trials Registry [ANZCTR] and ClinicalTrials.gov), with dates ranging from database inception through to 26^th^ January 2022. No limits to language were applied. Reference lists of included articles were also hand-searched for eligible studies. An example of the terms and syntax used in the MEDLINE search are presented in Supplementary file 2. All citations retrieved from the search were imported into Endnote™ (X9) bibliographic management software (for duplicate removal), and subsequently imported into Covidence (a systematic review management tool).

The title and abstract of each article were screened by one author (SG). Full-text articles were screened by two authors independently (SG and JW). Disagreements between the two authors were discussed with additional authors (AS and ML) and resolved as a group. Abstracts of articles not written in English were translated to English in order to assess eligibility.

Data were extracted using a customised data extraction form. The form collected information on study design, participants, sample size, setting, diagnosed reproductive condition(s), intervention (including duration and description), comparator, study outcomes and main findings (Table 1). The methodological quality of included studies was assessed using The Joanna Briggs Institute (JBI) critical appraisal tool for quasi-experimental studies. The purpose of the tool was to examine the extent to which included studies addressed potential biases in their design, conduct and analysis [31]. The methodological quality of the articles was assessed by two authors (SG and JW), independently. All studies presented evidence with high heterogeneity, therefore the body of literature was not amenable to meta-analysis and as such, the findings were presented as a narrative synthesis. The review protocol is registered with PROSPERO, ID: CRD42020196995.

**Table 1:** Data extraction table for group medical visits (GMV) in women’s health

**Author,** **Year,** **Country**	**Study design**	**Participants and sample**	**Setting**	**Condition**	**Duration and detail of intervention (GMV)**	**Outcomes of interest**	**Summary of findings**
1. Chao et al. 2015; USA [33]	Single arm pilot feasibility study	26 female participants 18yrs or older diagnosed with Chronic pelvic pain (CPP) by a health care provider	University-affiliated and public-hospital affiliated clinics	Chronic pelvic pain	Ten 2-h group medical visits held monthly for a total of ten months, among three cohorts of women Centering CPP – a program of integrative medicine group visits for women with CPP, based on the Centering model: healthcare assessment, education, and social support	**Reach:** Evaluation of patient representativeness Engagement evaluation **Effectiveness:** Health-related quality of life (HRQOL) SF36 Sexual Health Outcomes in Women Questionnaire (SHOW-Q) Patient Health Questionnaire (PHQ-9), Differential Emotions Scale (DES) Pain Catastrophising Scale (PCS) Measure Yourself Medical Outcomes Profile (MYMOP) **Implementation:** Feedback from facilitators and adherence with the curriculum **Adoption and Maintenance:** Percentage of centres that were were willing to implement the pilot program, and willingness to continue the program after the pilot study	**Reach:** Representativeness: Participants averaged 40 years of age (range 23–63 years) and were from diverse racial/ethnic backgrounds Half had graduated from college, and 76% had incomes of less than $50,000 **Engagement:** 15% did not attend any of the group visits. 73% attended 4 or more sessions **Effectiveness:** Improvements were observed for most measures of HRQOL (*n* = 16). The burden of CPP symptoms on HRQOL was reduced from 55.2 to 45.8 (*p* = .01) SF36 subscales for role limitations demonstrated statistically significant improvements. No differences were observed for subscales on physical functioning, emotional well-being, or general health **SHOW-Q:** Outcomes improved (30.5 to 50.3, *p* = .02) **MYMOP**: Outcomes were statistically significant on their overall MYMOP profile (4.2 to 3.1, *p* < .01) **Implementation:** High consistency was found across the group sessions (centres) Centering CPP was rated high for: education (94%); and using information in daily life (94%); emotional support (94%) and safety in discussing difficult issues (100%); a majority (88%) would recommend the group to other women with CPP **Adoption and maintenance:** 100% adoption rate of the program. All sites continued the program beyond the initial pilot study and provided resources to support the program ongoing
2. Harrison & Lach, 2017; USA [32]	Pilot study, quasi-experimental	11 participants non-pregnant, premenopausal females aged 18 years or older with a diagnosis of PCOS	University health clinic	Polycystic Ovary Syndrome	Group medical visit model, three visits each lasting 90–120 min held monthly for three consecutive months. Visits included measurement of vital signs; group interaction, education, and goal setting; and focused individual consultations	**Adherence** **Self efficacy:** Chronic Disease Self-Efficacy Scale **Health behaviour change:** diet and exercise behaviour **Patient satisfaction**	**Adherence:** 64% attended all 3 sessions **Self-efficacy:** Effect size using Cohen’s d showed a large effect size in 9 items (43%), a moderate effect size in 8 items (38%), and a small effect size in 3 items (14%). The 3-month post-intervention follow-up survey showed that these effects decreased overall but were still evident on several items **Health behaviour change**: over the course of the intervention, actual health practices did not change **Patient satisfaction**: participant expectations as “met” (*n* = 4, 44%) and “exceeded” (*n* = 5, 56%). Participant learning rated as “quite a bit” (*n* = 5, 56%) and “a great deal” (*n* = 4, 44%) Suggestions for improvement included longer duration, larger groups, blood testing, and more opportunity for participants to bond with each other
3. Prescott et al. 2016; USA [34]	Pilot program—feasibility	105 participants with a gynaecologic cancer scheduled to receive taxane and/or platinum-based chemotherapy	Tertiary academic medical centre	Gynaecol-ogical cancer	Shared Medical Appointment and Readiness Teaching (SMART) program for patients with gynaecologic cancer SMART was a one-off visit lasting two hours The group commences with an educational presentation delivered by a clinical pharmacist or advanced practice provider and is followed by a facilitated group discussion with the staffing physician providing a framework of social support	**Patient satisfaction:** Questionnaire including the Likert scale (questions 3–15), multiple choice and open-ended questions	**Length of appointment:** 95.6% reported ‘about right’ **Number of patients per group:** 94.29% reported ‘about right’ **Understood information provided (mean):** 4.73 + -0.05 **Adequate time to ask questions (mean):** 4.73 + -0.05 **Concerns about chemotherapy addressed (*n* = 104; mean):** 4.78 + -0.05 **Clear explanation of what to expect during chemotherapy (mean):** 4.84 + -0.05 **Interdisciplinary communication (mean):** 4.77 + -0.05 **SMAs are a good way of receiving information about chemo (*n* = 93; mean):** 4.65 + -0.08 **Other patients asked questions that I may not have asked (*n* = 92; mean):** 4.09 + -0.14 **Benefited from hearing the questions that other patients asked (*n* = 92; mean):** 4.33 + -0.12 **Would participate in other SMAs (*n* = 93; mean):** 4.31 + -0.01 **Would recommend SMAs to other patients (*n* = 94; mean):** 4.59 + -0.08 **Overall satisfaction with appointment (*n* = 94; mean):** 4.65 + -0.65 **Overall satisfaction with providers (*n* = 94; mean):** 4.79 + -0.05
4. Schneeberger et al. 2019; USA [35]	Quasi -experimental intervention study	21 participants Breast cancer survivors who have completed treatment, including those on hormonal therapy	Clinical setting	Breast cancer	‘‘Living Well after Breast Cancer’’ – a comprehensive lifestyle medicine intervention including seven 2-hou SMAs held every other week for a total duration of about 14 weeks The groups included a multidisciplinary lifestyle medicine team consisting of a physician, medical assistant, professional chef, yoga therapist, registered dietitian, and behavioural health specialist Each visit includes a physical exam and review of medications	**Biometric outcomes:** Weight BMI Body fat mass Lean body mass **Psychosocial metrics:** Perceived Stress Scale (PSS-4) Centre for Epidemiological Studies-Depression survey (CES-D 10) Patient Activation Measure (PAM) **Quality of life:** Patient-Reported Outcomes Measurement Information System (PROMIS-10) **The Block Dietary Fat Screener** **The Block Dietary Fruit, Vegetable, Fibre Screener** **End of program survey**	**Biometric outcomes (difference from visit 1 to visit 7):** Weight: *p* = < 0.01 BMI: *p* = < 0.01 Body fat mass: *p* = < 0.05 Lean body mass: *p* = < 0.01 Percent body fat: *p* = 0.22 Although more than half of patients decreased their percent body fat, three quarters of them also experienced undesired loss of lean body mass **Psychosocial metrics:** Perceived stress (*n* = 18): *p* = 0.73 Depression (*n* = 19): *p* = 0.14 Patient activation (*n* = 19): *p* = 0.10 Physical health (*n* = 18): *p* = 0.12 Mental health (*n* = 18): 0.87 Physical and Mental Health scores did not change significantly **Weekly dietary fat consumption** (*n* = 19): *p* = < 0.01 **Weekly dietary fruit, vegetable, fibre consumption** (*n* = 19): *p* = 0.62 **End of program survey:** One individual rated the intervention boring, and no one found it to be a waste of time 73% found the program educational 74% found the program enjoyable 36% reported the program exciting 46% described the program as life changing

## Results

**Fig. 1:**
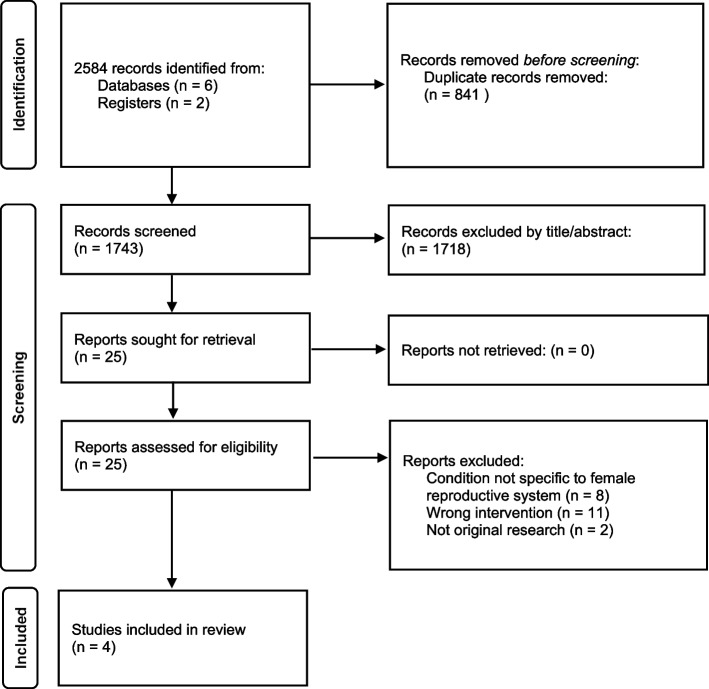
Flow diagram of study selection process

The search identified 2584 studies. A total of 841 duplicates were removed, leaving 1743 studies. The screening of titles and abstracts eliminated 1718 studies as they did not meet the review selection criteria. Of the remaining 25 studies selected for full-text analysis, 21 were excluded as they used group interventions not aligned with the SMA model (*n* = 11), included participants with a condition not specific to women or the female reproductive system (*n* = 8), or were not original research (*n* = 2). Supplementary file 3 provides on overview of articles excluded at full text screening. A total of four articles, published between 2015 and 2019, were included in the review. Figure 1 represents an overview of the article selection process.

All included studies were conducted in the United States. The studies comprised of a non-randomised, uncontrolled pilot study [32], two pilot feasibility trials [33, 34] and a non-randomised, uncontrolled feasibility study [35]. A total of 163 women participated in the four included studies and no studies including gender diverse participants were identified. Sample sizes ranged from 11 to 105 with a median sample size of 23.5. The characteristics of included studies are presented in Tables 1 and 2.

**Table 2:** Characteristics of included studies

		**Chao et al. 2015 **[33]** (Chronic pelvic pain)** ***n* = 26**	**Harrison & Lach 2017 **[32]** (PCOS)** ***n* = 11**	**Prescott et al. 2016 **[34]** (Gynaecological cancers)** ***n* = 105**	**Schneeberger et al. 2019 **[35]** (Breast cancer)** ***n* = 21**
**Study design**	Feasibility	x		x	x
	Pilot	x	x	x	
	Quasi-experimental				x
	Non-randomised & uncontrolled		x		x
**Feasibility outcomes**	Quantitative feasibility measures	x	x	x	x
	Qualitative feasibility feedback	x		x	
**Clinical outcomes**	Chronic Disease Self-Efficacy Scale		x		
	Biometric outcomes				x
	Psychosocial metrics				x
	Patient reported questionnaires (e.g. quality of life, pain scales)	x			x
	Dietary screening				x

Note: x indicates the design and outcome measures that apply to each study

The included studies used a diverse range of outcome measures and interventions, and investigated varied female reproductive conditions; accordingly, the studies were both clinically and methodologically heterogeneous. The included studies sampled women with breast cancer (*n* = 1 studies) [35], chronic pelvic pain (*n* = 1) [33], polycystic ovary syndrome (PCOS) (*n* = 1) [32], and varying gynaecologic cancers (*n* = 1) [34]. The interventions included IMGV (*n* = 1) [33] and GMVs (otherwise known as SMAs with terminology varying across studies) (*n* = 3) [32, 34, 35]. All included studies reported on feasibility and acceptability as outcomes, and all but one study [34] reported on clinical outcomes.

Critical appraisal using the JBI checklist for quasi-experimental studies indicated there was a clear identification of ‘cause’ and ‘effect’ across all studies, although no studies included a comparator group. Multiple measurements of study outcomes were used both pre- and post-intervention in all studies where this was applicable (*n* = 3). Outcomes were measured in a reliable way in all studies, and appropriate statistical analysis was used in all studies except one where this was not clear [34].

Findings are reported in two categories according to the objectives of the review: (1) feasibility and acceptability, and (2) clinical outcomes.

### Feasibility and acceptability

The included studies examined various aspects related to feasibility, including participant expectations, satisfaction [32, 34, 35], adoption and maintenance, implementation and reach [33]. All studies consistently found group visit interventions to be broadly feasible for, and well-accepted among women with female reproductive conditions.

Two studies investigated the feasibility of group visit interventions in women with a history of cancer. One study examined the feasibility of the “Living well after breast cancer” program in 21 breast cancer survivors [35]. The program – a SMA lifestyle medicine intervention – comprised seven 2-h SMAs held fortnightly for 14 weeks. The program included a multi-practitioner team, and consisted of nutrition, culinary medicine, physical activity and stress relief practices, as well as a physical exam and medication review at each visit. Feasibility was assessed using an end-of-program survey comprising a physician-generated list of responses including life-changing, educational, exciting, enjoyable, boring and a waste of time; 76% of respondents indicated the intervention was enjoyable, 46% described the program as life changing, and 36% found the program exciting.

Another study investigated the feasibility of the SMA and Readiness Teaching (SMART) program in 105 women with diverse gynaecologic cancers (including ovarian [36%], uterine [31%], cervical [15%], mullerian of unknown primary [13%], vaginal [4%] and vulvar cancer [1%]) who were scheduled to receive taxane and/or platinum-based chemotherapy [34]. The program comprised of a single 2-h session with a multi-disciplinary team of practitioners, including physicians, advanced practice providers, nurses, pharmacists, administrators and health education specialists, as well as a tour of the chemotherapy infusion facility and consumer health library. Ninety-five percent of patients were satisfied with the length of appointments and number of patients per group. Using a 5-point Likert satisfaction scale (with higher scores indicating greater satisfaction), the majority of patients indicated that they understood the information provided (4.73), had their concerns about chemotherapy addressed (4.78), were satisfied with interdisciplinary communication (4.77), had benefited from hearing questions from other patients (4.33), would recommend SMAs to other patients (4.59) and were satisfied overall with appointments (4.65) and providers (4.79).

A single study investigated the feasibility of IMGVs in 26 women with chronic pelvic pain [33]. The program comprised of healthcare assessments, education and social support, delivered across 2-h SMAs, which were held monthly for ten months [33]. Responses to measures of adoption and maintenance (percentage of women’s health centres that were approached and willing to implement the program), implementation (based on feedback from group facilitators and adherence to curriculum) and reach (measured by patient representativeness, recruitment success, participant attendance and participant feedback) indicated a high level of feasibility and acceptability. Of the 26 participants, 22 (73%) attended four or more sessions, and 4 (15%) did not attend any group visits. Reasons for missing sessions included pain, fatigue or feeling overwhelmed. Intervention fidelity was found to be high across group sessions, although stability of the group with respect to size was inconsistent (attendance ranged from 2–8 participants). The program was rated highly by participants in terms of providing education, using information in their daily lives, and providing a safe place to discuss difficult issues whilst being treated with respect (> 93% agreement across all aspects). The majority (88%) of women indicated they would recommend the group to other women with chronic pelvic pain. A 100% adoption rate was achieved from sites that were approached about implementing the program, with all sites continuing the program beyond the pilot.

The fourth study examined the feasibility of a SMA model in 11 patients with PCOS [32]. The GMVs were held monthly for three consecutive months and each group lasted 90–120 min in duration and was led by one health care provider/prescriber. The sessions included education, goal-setting and individual medical consultations (within the group setting). In this study, feasibility was measured using program adherence rates and quantitative survey feedback. All participants rated their expectations of the SMAs as “met” (44%) or “exceeded” (56%), with 64% attending all three sessions. All participants commented positively on the attainment of new knowledge, particularly the usefulness of the dietary information.

### Clinical outcomes

Three of the four included studies measured clinical outcomes, albeit using varied outcome measures, including a chronic disease self-efficacy scale [32]; patient-reported outcomes (e.g. Health related quality of life, pain catastrophising scale, sexual health outcomes, Measure Yourself Medical Outcomes Profile) [33]; and biometric (e.g. weight, BMI, body fat mass and lean body fat) and psychosocial outcomes (e.g. perceived stress, depression, mental health) [35].

One study examined the effectiveness of a SMA lifestyle medicine intervention on anthropometric and psychosocial outcomes in women with breast cancer. The study reported clinically significant improvements in participants’ mean weight (-4.89 pounds; *p* =  < 0.01), body mass index (-0.81; *p* =  < 0.01) and body fat mass (-2.58 pounds; *p* =  < 0.05) between visit one and visit seven [35]. The participant’s mean lean body mass also significantly declined over this period (-2.3 pounds; *p* =  < 0.01). Differences in body fat percentage and psychosocial outcomes (i.e. physical and mental health scores) between visit one and visit seven were not found to be statistically significant.

Another study examined the effectiveness of IMGVs on a range of clinical outcomes in women with chronic pelvic pain (CPP) [33]. The study reported a significant improvement in quality of life (*p* = 0.01) over the course of four or more IMGVs, including disease-specific quality of life (QoL; as measured by an adapted version of the Endometriosis Health Profile-5; with scores reducing from 55.2 to 45.8). No statistically significant differences in SF36 physical functioning, emotional well-being or general health subscales were observed over time. The number of unhealthy days reported in the previous month decreased by 6 days (*p* = 0.02). Sexual health outcomes also improved significantly over time, with a mean difference of 19.8 points from baseline to post-intervention on the Sexual Health Outcomes in Women Questionnaire (SHOW-Q) (*p* = 0.02). Women who attended 4 or more sessions also demonstrated a reduction in the severity of depressive symptoms (from 11.7 to 9.0 points on the Patient Health Questionnaire (PHQ-9); which represents a clinically relevant difference from minor depression to minimal symptoms [*p* =  < 0.02]). Women also demonstrated statistically significant improvements in symptom severity (4.6 to 3.4, *p* = 0.01), fewer limitations in activity (4.8 to 3.3, *p* =  < 0.01), and more optimal scores overall (4.2 to 3.1, *p* =  < 0.01) on the Measure Yourself Medical Outcome Profile (MYMOP), from baseline to post-intervention.

The third study investigated the effectiveness of a SMA model on the self-efficacy of patients living with PCOS [32]. Self-efficacy was measured at the first visit, third visit and three months after the final visit, using items from the Chronic Disease Self-efficacy Scale (CDSE). A large effect size (using Cohen’s d) was observed across nine items (43%) of the CDSE, including items relating to obtaining information about the disease, obtaining help from community, family and friends, disease management, symptom management and management of depression. A moderate effect size was reported across eight items (38%) of the CDSE, including items pertaining to regular exercise, communicating with healthcare providers, disease management and management of depression. A small effect size was observed for three items (14%) of the CDSE, including those relating to regular exercise and communication with healthcare providers. A decrease in overall self-efficacy was observed at 3-months follow-up post-intervention, but was still evident for several items. Health behaviour practices did not change significantly over the course of the intervention.

## Discussion

This review is the first to analyse the best available evidence of the effectiveness, feasibility and acceptability of group medical and allied health interventions for women’s health conditions. Focusing only on group interventions that are traditionally delivered via one-on-one consultation (rather than interventions usually delivered in groups such as yoga or group psychotherapy), the review revealed a dearth of research in this area. Notwithstanding, the findings align with nominal research on SMAs for non-gender specific chronic disease management conducted in other settings [5, 8, 36]. As the majority of studies included in the review were pilot feasibility studies, and not all studies reported on clinical outcomes, the hypotheses that can be drawn from the available evidence have a notable emphasis on the feasibility and acceptability of group medical interventions, rather than effectiveness.

The studies in this review suggest group visits may be a feasible approach to managing women’s health conditions. All four included studies reported high levels of patient satisfaction, with participants indicating that their expectations of the SMAs had been met or exceeded, and that they would recommend the group to other patients. The one study examining adoption and maintenance of an IMGV reported a 100% adoption rate amongst providers offering women’s health-focused services, albeit across a small number of settings [33]. Reports of positive provider satisfaction corroborate findings from other group visit research in non-gender-specific populations [3, 5].

These overall findings on feasibility are broadly supported by other studies on SMAs [8], where group visits have been found to be feasible, acceptable, cost-effective and time-efficient in diverse populations [5, 11, 37]. Two of the four included studies incorporated either an integrative medicine component in the intervention or lifestyle medicine (e.g. culinary medicine, yoga) [33, 35], suggesting the IMGV subset of SMAs may provide a practicable method for integrating evidence-based complementary therapies and lifestyle medicine into patient care. This has implications for the management of female reproductive conditions – for example endometriosis (often characterised by CPP), where conventional medicine alone does not meet the health care needs of those living with the condition and a multi-disciplinary approach is recommended [24]. While IMGVs may be a more effective way to support patients with such conditions by proffering an effective model for inclusion of multidisciplinary services and patient education into routine care (via improved efficiency of primary care delivery), further research is needed to investigate their use.

In addition to the evidence supporting group visits for non-gender specific chronic conditions, research examining group visits for preconception and prenatal care also demonstrate promising results in terms of both efficacy and feasibility. For example, in the area of maternal health, research findings have typically favoured group care over one-on-one care for birth outcomes and clinical parameters in conditions such as gestational diabetes mellitus [38], thus complementing the findings of this review. While there is limited research examining the effectiveness of group visit interventions for female reproductive conditions, current evidence supporting group visits for other areas of women’s health (such as group prenatal care) provides a solid basis for proposing larger and longer-term studies of group visits for female-specific conditions.

Despite inconclusive evidence for the clinical efficacy of group visits for female reproductive conditions, some noteworthy changes in clinical outcomes were observed (albeit in small studies) for conditions such as CPP and PCOS. The promising clinical results observed in the study of IMGVs for CPP [33] complements prior research highlighting the limitations of gynaecological treatments for CPP, suggesting a multidisciplinary approach may help improve the management of this condition [39]. Furthermore, improvements in self-efficacy suggest group-based models of care may be helpful in addressing the needs of some patients living with a diagnosis of PCOS. Interestingly, patients in the aforementioned study [32] suggested continuing the group-based care intervention for at least a year, indicating that the duration of the intervention may not have been sufficient, which may account for the lack of health behaviour change reported by patients. Similarly, research investigating group visits in other populations has shown patients prefer, and demonstrate better clinical outcomes, when exposed to more frequent visits and longer intervention timeframes [5, 33, 40]. Thus, future research of group-based care should consider using longer intervention periods, more frequent group meetings and larger sample sizes. We suggest future research also needs to be conducted to investigate the efficacy of group medical visits with analysis for each different population living with a female reproductive condition and their unique healthcare needs. Although no known adverse events have been associated with group visits in the literature, one study in this review hypothesised that the personal nature of conditions such as CPP may serve as a hindrance for some people to participate. It is possible that this hypothesis may also apply to people with other gynaecological conditions, adding to the case for further research.

There are several limitations to this review. As the review comprised of mostly quasi-experimental pilot and feasibility studies, and the sample sizes were small, the generalisability of the review findings are somewhat limited. The broad nature of the review and heterogeneity of studies also meant that meta-analysis of the data was not possible; instead, the data were presented as a narrative synthesis. Although a limitation in part, this also represents a strength of the review as narrative syntheses (relative to meta-analyses) allow for deeper analysis of meaning and interpretation [41]. Given the studies did not utilise control groups, and only one study included a comparison group, it is unknown how the effect of the intervention compares with usual (one-on-one) care. Further, all included studies were conducted in the USA – where heterogeneous private and public insurance measures and funding models may better support group care – and thus, may not readily translate to other settings affected by different social, economic or health parameters. Despite these limitations, group visits for female reproductive conditions appear to be feasible; thus more clinical trials addressing previous limitations are encouraged.

## Conclusion

This review provides insight into the feasibility and acceptability of group visits for the management of female reproductive conditions. Given the small number and methodological limitations of studies included in this review, no conclusions regarding the clinical effectiveness of group visits for female-specific conditions (relative to one-on-one consultations) can be drawn from this evidence. However, the evidence suggests that group interventions may be feasible and acceptable to women with female reproductive conditions such as breast cancer, gynaecological cancer, CPP and PCOS. Accordingly, group visit interventions, such as SMAs and IMGVs, may be an untapped model of care for people living with female reproductive conditions, particularly where conventional models are not meeting the health care needs of people living with these conditions. Given the evidence of effectiveness, acceptability and reach for group visits in other populations, further research investigating the effectiveness and feasibility of group-based integrative women’s health interventions is justified.

## Supplementary Information

**Additional file 1. **PRISMA checklist.

**Additional file 2: Supplementary File 2.** Search terms and syntax.

**Additional file 3: Supplementary File 3.** Studies excluded at full text (*n* = 20).

## Data Availability

Data will be made available upon reasonable request. Please contact corresponding author SG.

## Abbreviations

CDSE: Chronic Disease Self-efficacy Scale
CPP: Chronic Pelvic Pain
GDM: Gestational Diabetes Mellitus
GMV: Group Medical Visit
IMGV: Integrative Medicine Group Visit
MYMOP: Measure Yourself Medical Outcome Profile
PCOS: Polycystic Ovary Syndrome
PHQ-9: Patient Health Questionnaire
SHOW-Q: Sexual Health Outcomes in Women Questionnaire
SMA: Shared Medical Appointment
