# Supplementary material for: Feasibility and efficacy of implementing group visits for women’s health conditions: a systematic review
Source: BMC Health Serv Res. 2023 May 26;23:549. doi: 10.1186/s12913-023-09582-6 (PMC10214697; doi:10.1186/s12913-023-09582-6)
Supplement: Supplementary file 2 — Additional file 2: Supplementary File 2. Search terms and syntax. [file 12913_2023_9582_MOESM2_ESM.docx]

**Table S2. Search terms and syntax**

| Medline (Ovid) | Shared medical appointments/ OR group processes/ OR group medical care.tw OR group medical appointment*.tw OR group medical meeting*.tw OR group medical visit*.tw OR group medical clinic*.tw OR group clinic.tw OR group meeting*.tw OR group care.tw OR group appointment.tw OR group visit*.tw OR shared medical visit*.tw OR shared medical appointment*.tw OR cluster visit*.tw OR group outpatient visit*.tw OR group outpatient clinic*.tw OR group outpatient appointment*.tw OR group outpatient meeting*.tw OR group outpatient care.tw AND exp women’s health/ OR women’s health services/ OR women/ OR pregnancy/ OR pregnancy complications/ OR exp menstruation disturbances/ OR amenorrhea/ OR dysmenorrhea/ OR menorrhagia/ OR oligomenorrhea/ OR premenstrual syndrome/ OR pelvic pain/ OR endometriosis/ OR fibroma/ OR leiomyoma/ OR hirsutism/ OR polycystic ovary syndrome/ OR ovarian cysts/ OR myoma/ OR metrorrhagia/ OR reproductive health/ OR (acne vulgaris/ AND female/) OR (women* AND health).tw OR metrorrhag*.tw OR endometrial hyperplasia.tw OR maternal depression.tw OR pelvic pain.tw OR polycystic ovar*.tw OR endometriosis.tw OR adenomy*.tw OR fibroid.tw OR fibroma*.tw OR myoma.tw OR menstrual pain.tw OR period pain.tw OR menstruation disorder.tw OR dysmenorrh*.tw OR amenorrh*.tw OR oligomenorrh*.tw OR oligo amenorrh*.tw OR hirsute*.tw OR premenstrual*.tw OR pre menstrua*.tw OR leiomyoma*.tw OR menorrhag*.tw OR heavy menstua*.tw OR menopause.tw OR cervical cancer.tw OR breast cancer.tw OR gestational hypertension.tw OR gestational diabetes.tw OR pregnan*.tw (limit to human) |
| --- | --- |
