# Supplementary material for: Feasibility and efficacy of implementing group visits for women’s health conditions: a systematic review
Source: BMC Health Serv Res. 2023 May 26;23:549. doi: 10.1186/s12913-023-09582-6 (PMC10214697; doi:10.1186/s12913-023-09582-6)
Supplement: Supplementary file 3 — Additional file 3: Supplementary File 3. Studies excluded at full text (n = 20). [file 12913_2023_9582_MOESM3_ESM.docx]

**Supplementary File 3**

**Studies excluded at full text (*n* = 20)**

| **Study** | **Title** | **Reason for exclusion** |
| --- | --- | --- |
| Thacker, Maxwell & Saporito, 2005 | Shared medical appointments: Facilitating interdisciplinary care for midlife women | Condition not specific to women (*n* = 1) |
| Balabanovic, Ayers & Hunter, 2012 | Women's experiences of Group Cognitive Behaviour Therapy for hot flushes and night sweats following breast cancer treatment: an interpretative phenomenological analysis | Condition not specific to female reproductive system  (*n* = 7) |
| Mazzoni, Hill, Webster et al., 2016 | Group prenatal care for women with gestational diabetes |  |
| Nasso, McCloskey, Nordquist et al., 2018 | The Gestational Diabetes Group Program |  |
| Parikh, Jelin, Iqbal et al., 2017 | Glycemic control, compliance, and satisfaction for diabetic gravidas in centering group care |  |
| Potter, Duthely, Diaz-Mendez et al., 2019 | Implementing CenteringPregnancy Group Prenatal Care for Minority Women Living with HIV at an Urban University Hospital |  |
| Raymond, Foureur & Davis, 2014 | Gestational weight change in women attending a group antenatal program aimed at addressing obesity in pregnancy in New South Wales, Australia |  |
| Thomas, Komiti & Judd et al., 2014 | Pilot early intervention antenatal group program for pregnant women with anxiety and depression |  |
| Brunet & St-Aubin, 2016 | Fostering positive experiences of group-based exercise classes after breast cancer: what do women have to say? | Wrong intervention  (*n* = 10) |
| Emslie, Whyte, Campbell et al., 2007 | 'I wouldn't have been interested in just sitting round a table talking about cancer'; exploring the experiences of women with breast cancer in a group exercise trial |  |
| Guillet, Cirino, Hart et al., 2019 | Mindfulness-Based Group Cognitive Behavior Therapy for Provoked Localized Vulvodynia: A Randomized Controlled Trial |  |
| Huang, Rowen, Abercrombie et al., 2017 | Development and Feasibility of a Group-Based Therapeutic Yoga Program for Women with Chronic Pelvic Pain |  |
| Kaaya, Blander, Gretchen et al., 2013 | Randomized controlled trial evaluating the effect of an interactive group counselling intervention for HIV-positive women on prenatal depression and disclosure of HIV status |  |
| Kolden, Strauman, Ward et al., 2002 | A pilot study of group exercise training (GET) for women with primary breast cancer: feasibility and health benefits |  |
| Leach, Covington, Voss et al., 2019 | Effect of Group Dynamics- Based Exercise Versus Personal Training in Breast Cancer Survivors |  |
| Lim, Versace, Janus et al., 2017 | Comparing a telephone- and a group-delivered diabetes prevention program: Characteristics of engaged and non-engaged postpartum mothers with a history of gestational diabetes |  |
| Mann, Smith, Hellier et al., 2012 | Cognitive behavioural treatment for women who have menopausal symptoms after breast cancer treatment (MENOS 1): a randomised controlled trial |  |
| Rissanen, Arving, Ahlgren et al., 2014 | Group versus individual stress management intervention in breast cancer patients for fatigue and emotional reactivity: A randomised intervention study |  |
| Chao, Abercrombie & Duncan, 2012 | Centering as a Model for Group Visits Among Women with Chronic Pelvic Pain | Not original research  (*n* = 2) |
| Nguyen, Allen, Gorman et al., 2014 | Group prenatal care for women with pre-gestational type II diabetes mellitus: A cost-effectiveness analysis |  |
